# Endoplasmic reticulum stress in skeletal muscle dysfunction of type 2 diabetes: mechanisms and therapeutic implications

**DOI:** 10.3389/fendo.2026.1769545

**Published:** 2026-02-13

**Authors:** Xin Wang, Mingdi Li, Zengpeng Chi, Mingshan Wang, Jiayi Liu, Ailin Li, Bailin Song, Le Tong

**Affiliations:** 1 College of Acupuncture and Tuina, Changchun University of Chinese Medicine, Changchun, Jilin, China; 2 Preventive and Health Care Department, Qingdao Hiser Hospital Affiliated to Qingdao University (Qingdao Traditional Chinese Medicine Hospital), Qingdao, Shandong, China; 3 Department of Stomatology, Qingdao West Coast New District Central Hospital, Qingdao, Shandong, China; 4 Department of Clinical Medicine, Shandong Second Medical University, Weifang, Shandong, China; 5 Department of Tuina, Shenzhen Hospital of Integrated Traditional Chinese and Western Medicine, Shenzhen, Guangdong, China; 6 Department of Traditional Chinese Medicine, Affiliated Hospital of Shandong Second Medical University, Weifang, Shandong, China; 7 Department of Acupuncture, Qingdao Hiser Hospital Affiliated to Qingdao University (Qingdao Traditional Chinese Medicine Hospital), Qingdao, Shandong, China

**Keywords:** endoplasmic reticulum stress, insulin resistance, muscle atrophy, therapeutic strategies, type 2 diabetes

## Abstract

With the continuous rise in the global prevalence of type II diabetes mellitus (T2DM), the associated skeletal muscle complications, including insulin resistance, muscle atrophy, and physical frailty, have garnered increasing attention. Skeletal muscle plays a vital role as a metabolic and endocrine organ and is considered a key factor in the pathological mechanisms of T2DM. Despite significant advances in understanding, the intrinsic molecular mechanisms underlying skeletal muscle dysfunction remain incompletely elucidated. Recent studies have highlighted a significant cellular stress response known as endoplasmic reticulum stress (ERS), which is triggered by hyperglycemia, lipotoxicity, and inflammation, and may serve as a pivotal hub in T2DM pathology. This review narratively examines published articles from the past five years, focusing on experimental studies related to ERS and T2DM myopathy. Comprehensive searches were conducted in electronic databases for journal articles published between 2020 and 2025.This review synthesizes experimental studies to elucidate how ERS disrupts muscle homeostasis via the unfolded protein response (UPR) pathways (PERK, IRE1α, and ATF6) contributing to insulin resistance and activation of protein degradation systems. Consequently, intervention strategies targeting ERS may offer new insights and directions for the prevention and treatment of T2DM-related muscle disorders. This review aims to explore the mechanisms by which ERS contributes to T2DM myopathy, identifying potential therapeutic targets and providing a foundation for future clinical research.

## 1 Introduction

The continuous rise in the global prevalence of type II diabetes mellitus (T2DM) has made its skeletal muscle complications an increasingly severe clinical challenge (1–3). Progressive insulin resistance, reduction in muscle mass, and progressive functional decline collectively constitute key factors affecting patients’ quality of life and clinical prognosis (4). Skeletal muscle plays a central role in maintaining systemic glucose homeostasis. It characterized as the primary insulin-sensitive tissue which responsible for approximately 80% of postprandial glucose disposal. It also functioned as an endocrine organ that participates in systemic metabolic regulation through the secretion of myokines (5, 6).

In the pathological context of T2DM, skeletal muscle is chronically exposed to multiple stressors including hyperglycemia, lipotoxicity, and chronic low-grade inflammation (7). These pathological factors collectively drive two interrelated processes: insulin resistance and diabetic sarcopenia (8). The former manifests as dysfunction of metabolic pathways, while the latter is characterized by progressive loss of muscle structure and contractile function, with both synergistically accelerating the overall decline in patient’s physical function. Although previous studies have predominantly focused on the roles of mitochondrial dysfunction and inflammatory signaling pathways in T2DM myopathy, the current understanding of the upstream regulatory mechanisms coordinating these pathological processes remains incomplete.

In recent years, endoplasmic reticulum stress (ERS) has increasingly emerged as a pivotal link integrating various metabolic abnormalities, highlighting its significance in the research of T2DM myopathy mechanisms (9, 10). The endoplasmic reticulum (ER), as a key organelle responsible for protein synthesis, folding, modification, and calcium homeostasis maintenance, is highly susceptible to disruption in the pathological environment of T2DM, leading to the accumulation of unfolded or misfolded proteins and subsequently triggering ERS (11). The subsequently activated unfolded protein response (UPR) attempts to restore protein homeostasis through three core signaling pathways which are PERK, IRE1α, and ATF6 (12, 13). However, prolonged or excessive ERS will prompt the UPR to shift from adaptive responses to the promotion of insulin signaling suppression and the activation of protein degradation systems, ultimately forming a vicious cycle in which insulin resistance and muscle atrophy exacerbate each other (14–17).

It is noteworthy that ERS is not isolated in T2DM myopathy. It engages in extensive cross-talk with pathological processes such as oxidative stress and autophagy dysregulation, forming a complex regulatory network that collaboratively drives disease progression (18). The characteristic of this multi-pathway interaction makes ERS a crucial entry point for elucidating the pathological mechanisms of skeletal muscle in T2DM.

This article aims to systematically elucidate the core mechanisms by which ERS mediates the evolution of skeletal muscle from metabolic disorder to structural atrophy in the context of T2DM. By analyzing the induction pathways of ERS in the diabetic muscle microenvironment, we clarify the specific roles of various UPR signaling pathways in insulin resistance and muscle atrophy. Furthermore, we explain the networked interactions between ERS and other pathological processes, with the expectation of providing a theoretical framework for the development of novel prevention and treatment strategies for muscle complications associated with T2DM.

## 2 Methods

A comprehensive search strategy was developed in collaboration with a medical librarian to ensure thoroughness and precision. We systematically integrated various search terms and Medical Subject Headings (MeSH) related to T2DM, ERS, and skeletal muscle into a tailored search protocol specifically designed for PubMed. This protocol was subsequently adapted for application in other relevant databases. Searches were conducted across multiple platforms including PubMed, Cochrane Library, CINAHL, Web of Science, and CNKI, targeting journal articles pertaining to experimental studies published within the last five years (2020-2025), with no restrictions on language.

In addition to database searches, we manually reviewed the reference lists of selected articles to identify other potentially relevant studies. For instances where full text articles were inaccessible, we reached out to the corresponding authors via email to request copies. The eligibility of titles and abstracts obtained from the search results was independently assessed by two reviewers (XW and ML). Any disagreements between the reviewers were resolved through consensus or by consulting a third reviewer (LT) to ensure the integrity of the selection process.

## 3 Triggers of ERS in skeletal muscle of T2DM

### 3.1 Nutritional excess and metabolic substrate toxicity

In the pathological state of T2DM, the emergence of ERS is closely linked to nutrient overload and the toxicity of metabolic substrates. When blood glucose levels rise, advanced glycation end products (AGEs) trigger ERS by generating reactive oxygen species (ROS). Clinical studies have confirmed that the levels of AGEs in the serum of T2DM patients are significantly higher than those in healthy individuals, and they exhibit a significant positive correlation with fasting plasma glucose (FPG), glycated hemoglobin (HbA1c), and the homeostasis model assessment of insulin resistance (HOMA-IR) (19). In this process, the accumulation of AGEs not only impairs cellular functions but also exacerbates diabetes-related complications by increasing the burden on the ER.

Recent studies have further revealed the critical signaling nodes connecting AGEs, ER stress, and insulin resistance: the GRP78 protein, a core marker of ER stress, is significantly elevated in the circulation of patients with T2DM and is directly positively correlated with HbA1c and AGEs concentrations. Meanwhile, the intracellular pseudo-kinase tribbles homolog 3(TRB3), induced by ER stress, is also abnormally expressed in the serum of these patients. It is not only positively correlated with all glucose indicators and insulin resistance but also shows significant positive correlations with both AGEs and GRP78 levels (20). In a diabetic rat model, AGEs have been shown to mediate β-cell apoptosis by inducing the expression of TRB3 (21, 22). These research findings are consistent with the results of studies showing an increase in TRB3 in the liver of obese insulin-resistant patients and in the skeletal muscle of patients with T2DM (23, 24). Animal experiments have shown that the specific overexpression of TRB3 in the liver of mice inhibits the insulin signaling pathway, leading to increased hepatic glucose output and hyperglycemia (25). Conversely, in a rat model of T2DM, the use of gene silencing technology to knock down TRB3 effectively improves insulin resistance and myocardial injury (26, 27). Overexpression of GRP78 in pancreatic β-cells has been shown to improve glucose intolerance and insulin resistance induced by a high-fat diet, and to protect β-cells from lipotoxicity (28). These findings indicated that the accumulation of AGEs may exacerbate the condition by regulating this signaling pathway, thereby promoting inflammation and stress responses.

Free fatty acids, particularly palmitic acid, are considered potent inducers of ER stress. It can upregulate molecules associated with ER stress, such as PDIA4, BIP, and ATF4, thereby exacerbating the metabolic burden and dysfunction of muscle. Existing studies have indicated that palmitic acid-induced ER stress is related to the dysregulation of protein folding in muscle cells, which in turn affects the physiological functions of the cells (29). Palmitic acid (PA) specifically upregulates the expression of the ER oxidoreductase ERO-1αin pancreatic β-cells (30). The activation of ERO-1α leads to the accumulation of its byproduct hydrogen peroxide in the ER lumen, which triggers oxidative ER stress, disrupts calcium homeostasis, and causes mitochondrial dysfunction, ultimately driving β-cell apoptosis (31). The findings suggest that the knockout of ERO-1α can significantly alleviate the toxicity of palmitic acid, whereas its over expression exacerbates cell death (32). This process is closely related to classical ER stress apoptotic pathways, such as C/EBP homologous protein (CHOP).

### 3.2 Exacerbation of inflammation and oxidative stress

Chronic low-grade inflammation is a common phenomenon in T2DM, driven by the activation of immune cells in adipose tissue, liver, and pancreatic islets, such as macrophages, which release cytokines including tumor necrosis factor (TNF) -α, interleukin (IL)-1β, and IL-6 (33). Among these, pancreatic β-cells are particularly sensitive to IL-1β due to the high expression of IL-1 receptors, and prolonged exposure can directly lead to their functional disruption and apoptosis (34, 35). These inflammatory factors not only interfere with insulin signaling pathways but also disrupt cellular homeostasis by activating pathways (e.g. JNK and nuclear factor kappa B [NF-κB]), exacerbating ERS and oxidative stress (36, 37). In skeletal muscle, studies have clearly revealed that factors like Asprosin can activate PKCδ, synergistically initiating the NF-κB inflammatory pathway and ERS, collectively leading to insulin resistance (38). A recent study indicates that environmental pollutants such as per- and polyfluoroalkyl substances (PFAS) can induce a chronic inflammatory state by upregulating pro-inflammatory cytokine expression, thereby activating ERS pathways, promoting β-cell apoptosis and insulin resistance (39). This process, along with lipotoxicity and glucotoxicity, constitutes the core of metabolic stress.

There exists a profound interaction between oxidative stress and ER stress, forming a vicious cycle. The generation of ROS not only directly damages cellular structures but also exacerbates the pathological state of T2DM by activating ER stress sensors such as PERK and IRE1α (40). Conversely, the persistence of ER stress can lead to mitochondrial dysfunction, resulting in the production of more ROS. In a high-fat diet combined with STZ-induced T2DM rat model, the levels of lipid peroxides (LPO) in pancreatic tissue were significantly elevated, while the levels of antioxidant enzymes (SOD, CAT, GPx) and reduced glutathione (GSH) decreased. This was accompanied by an upregulation of ER stress-related apoptotic proteins such as CHOP, Caspase-12, and Caspase-3, clearly demonstrating the synergistic role of these two stresses in promoting β-cell exhaustion (41). Autophagy, as a key mechanism for the clearance of damaged components (e.g. dysfunctional mitochondria), is often impaired under the triple pressure of inflammation, oxidative stress, and ER stress, further accelerating the dysfunction of β-cells (42–44). In β-cells, mitophagy mediated by the PINK1/Parkin pathway is crucial for alleviating oxidative stress (45, 46); while ER stress regulates autophagic activity through its key sensors PERK, ATF6, and IRE1α to maintain homeostasis (47).

### 3.3 Calcium homeostasis disorder

In the context of T2DM, oxidative stress negatively impacts the functionality of the sarco(endo)plasmic reticulum Ca^2+^-ATPase (SERCA), leading to a disruption of ER calcium ion balance and consequently triggering ERS (48). The core mechanism involves the oxidative environment within the endoplasmic reticulum lumen directly damaging the function of calcium handling proteins such as SERCA, while also potentially activating calcium release channels like inositol trisphosphate receptor (IP3R), which together result in the depletion of the ER calcium store and abnormal fluctuations in cytosolic calcium concentrations (49). Calcium ions play a crucial role in cellular signal transduction and muscle contraction, and dysregulation of calcium homeostasis can lead to a decline in muscle cell function (50). Further research reveals that this dysregulation of calcium homeostasis and ERS can form a vicious cycle: calcium ion imbalance is an initial factor triggering ERS, while persistent ERS can further exacerbate oxidative stress and the disruption of calcium homeostasis through its downstream signals, including the activation of the CHOP pathway (51).

**Figure 1:**
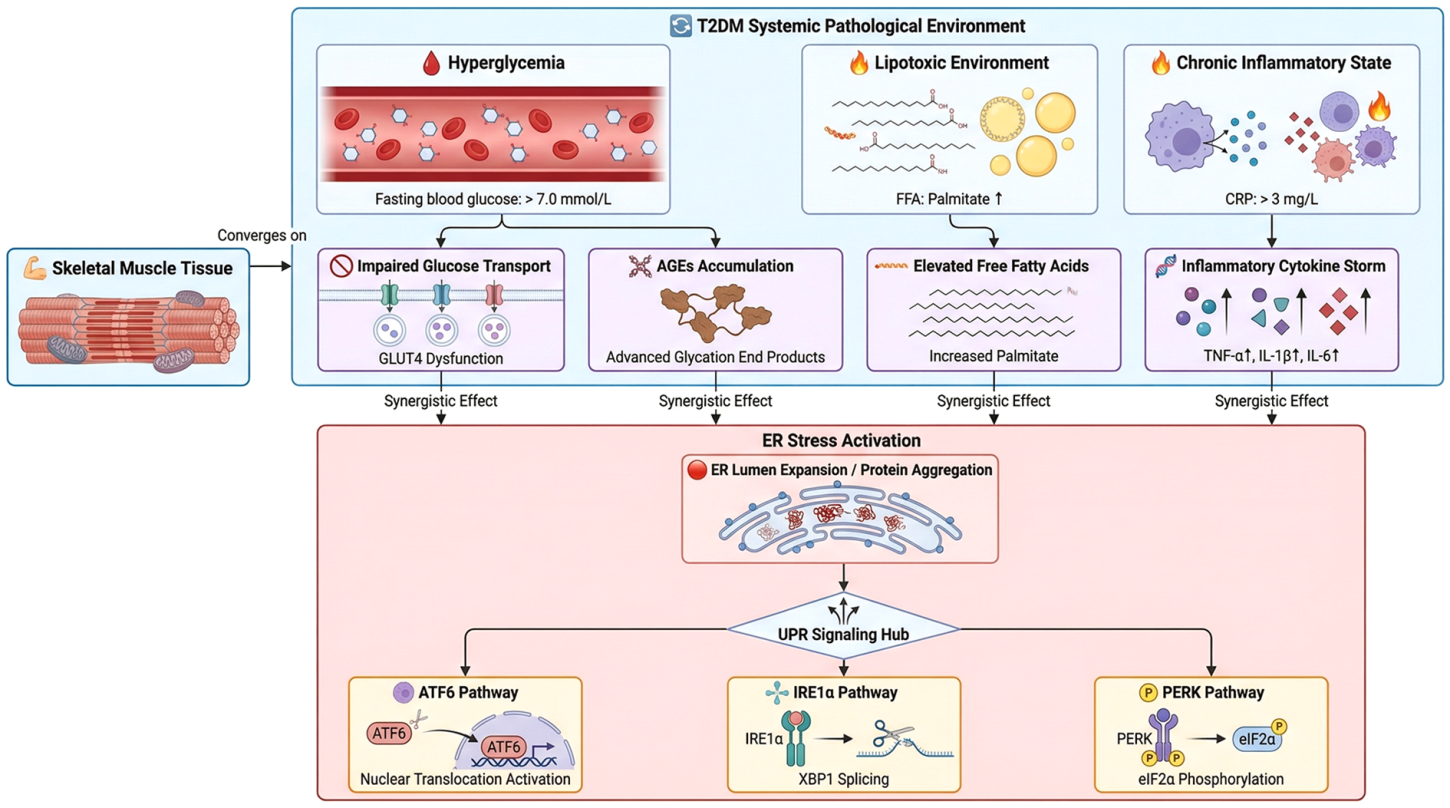
ERS and UPR signaling in T2DM skeletal muscle. This schematic outlines how T2DM-associated hyperglycemia (fasting glucose >7.0 mmol/L), lipotoxicity (elevated palmitate), and chronic inflammation (CRP >3 mg/L) converge in skeletal muscle to induce GLUT4 dysfunction, AGEs accumulation, palmitate overload, and cytokine storm (TNF-α, IL-1β, IL-6). These insults trigger ERS (ER lumen expansion/protein aggregation), activating the UPR via three branches: (1) PERK: BiPdissociation → PERK autophosphorylation → eIF2α phosphorylation → ATF4/CHOP induction (driving oxidative stress/apoptosis); (2) IRE1α: Oligomerization → uXBP1 splicing (sXBP1 for ERAD/chaperones) + RIDD; TRAF2/ASK1 recruitment → JNK/p38 MAPK (apoptosis via CHOP/Bcl2); (3) ATF6: BiPdissociation → Golgi S1P/S2P cleavage → nuclear translocation (ERQC gene induction).

Research indicates that the disruption of calcium homeostasis not only exacerbates ER stress but may also further promote muscle atrophy associated with T2DM by affecting the metabolism and survival of muscle cells (52). This pathway has been well-established in pancreatic β-cells: the disruption of calcium homeostasis caused by ER stress directly impairs insulin synthesis and secretion, and activates pro-apoptotic pathways (e.g. CHOP and JNK), ultimately leading to cellular functional decline and death (53). This suggests that in T2DM, an ER stress pathway initiated by oxidative stress, mediated by calcium homeostasis disruption, and ultimately leading to cellular dysfunction, may be a common mechanism driving various tissue lesions (**Figure 1**).

## 4 ER homeostasis imbalance and its related target proteins

SR is a highly specialized form of the endoplasmic reticulum found in skeletal muscle cells, characterized by an extensive network of small tubules (54). This structural and functional specialization means that the ERS encountered in T2DM skeletal muscle operates within a unique context. The SR’s primary role is calcium cycling, enabled by a specific proteome featuring isoforms like SERCA1a and ryanodine receptor 1 (55, 56). Metabolic insults such as lipotoxicity are particularly damaging to this precise system in T2DM. For example, oxidative stress can directly inhibit SERCA activity or promote RyR dysfunction, leading to SR calcium leak (57). This local calcium dyshomeostasis acts as a potent inducer of ERS and simultaneously disrupts excitation-contraction coupling, the core function of muscle (54). Furthermore, elevated cytosolic calcium can activate muscle-specific proteolytic pathways (e.g., involving calpains), creating a direct mechanistic link from ERS to atrophy that is amplified in this tissue. Therefore, the UPR in diabetic muscle cannot be fully understood in isolation; it is intrinsically linked to the failure of this specialized calcium-handling apparatus. The following sections detailing the PERK, IRE1α, and ATF6 pathways should be interpreted through this essential lens of muscle-specific pathophysiology. This core membranous organelle not only dominates muscle contraction by regulating calcium ion homeostasis but also serves as the center for protein synthesis, folding, processing, and secretion. The stability of its internal environment is crucial for maintaining the metabolism and structural integrity of skeletal muscle (58–60). Persistent hyperglycemia, excess free fatty acids, and chronic inflammation together constitute a source of cellular stress, leading to the accumulation of unfolded or misfolded proteins within the ER, thereby triggering ERS (61). In response to this crisis, cells initiate the highly conserved UPR. This response is primarily mediated by the key molecular chaperone BiP/GRP78 located in the lumen of the ER. Under normal conditions, BiP binds to and inhibits three transmembrane sensors: PERK, IRE1α, and ATF6. However, during ERS, BiP dissociates from these sensors and instead binds to misfolded proteins, leading to the activation of three parallel signaling pathways involved in the unfolded protein response (62, 63).

The initial activation of the UPR is an adaptive protective program aimed at restoring ER homeostasis. It works by transiently suppressing global protein translation (to reduce the load of new proteins) and transcriptionally upregulating ER chaperones, folding enzymes, and components of ER-associated degradation (to enhance the folding and quality control capacity of the ER), which collaboratively alleviate ER stress (64, 65). However, in the chronic progression of diabetes, persistent and unrelenting ER stress can lead to an irreversible shift of the UPR from an adaptive response to a pathological output (66). This decompensated UPR signaling directly interferes with insulin signaling through its downstream pathways, for instance, by promoting the degradation of insulin receptor substrate 1 (IRS-1) or rendering it functionally inactive, thus becoming a key driver of insulin resistance in skeletal muscle (67, 68). Meanwhile, sustained ERS activates apoptotic signaling and upregulates muscle-specific ubiquitin ligases, collaboratively driving protein degradation and cell loss in muscle fibers, ultimately resulting in diabetic muscle wasting (69). Therefore, the maintenance of ER homeostasis is a decisive factor for skeletal muscle to preserve metabolic sensitivity and structural integrity in a diabetic environment. The following sections will detail the specific mechanisms of the three core UPR signaling pathways: PERK, IRE1α, and ATF6, in the processes of insulin resistance and skeletal muscle atrophy in diabetes.

### 4.1 PERK

PERK, as a core sensor of ER stress, plays a pivotal role in the pathological process of diabetic skeletal muscle. Under diabetic conditions, metabolic stresses such as hyperglycemia and lipotoxicity lead to ER dysfunction, causing PERK to dissociate from the molecular chaperone BiP and undergo oligomerization and autophosphorylation (70, 71). In skeletal muscle tissues of STZ-induced diabetic rats or db/db mice, a significant increase in PERK phosphorylation levels can be observed through Western Blot analysis (72). Activated PERK subsequently phosphorylates its downstream substrate eukaryotic translation initiation factor 2 alpha (eIF2α), which not only globally inhibits protein translation but also selectively upregulates the translation of activating transcription factor 4 (ATF4) (73). ATF4, in turn, drives the expression of CHOP, which has been shown to promote the ubiquitin-mediated degradation of IRS-1 and induce the expression of inflammatory factors, thereby exacerbating insulin signaling pathway resistance through a negative feedback mechanism (74, 75). Studies indicate that inducing ERS in C2C12 myotubes using PA and employing CHOP siRNA for knockdown can validate this; if the knockdown of CHOP alleviates PA-induced IRS-1 degradation and Akt phosphorylation inhibition, it directly demonstrates the causal role of CHOP in this process (76).

The sustained activation of the PERK/eIF2α/ATF4 signaling axis is directly involved in the regulation of diabetic muscle atrophy. This pathway transcriptionally upregulates the expression of the key E3 ubiquitin ligase Atrogin-1 associated with muscle atrophy, and collaborates to activate the ubiquitin-proteasome system, accelerating the degradation of myofibrillar proteins (77). Studies have confirmed that in skeletal muscle of diabetic models, the activation of the PERK pathway is closely related to elevated levels of Atrogin-1, accumulation of ubiquitinated proteins, and a reduction in muscle fiber cross-sectional area. The use of a PERK inhibitor or the chemical chaperone 4-phenylbutyric acid (PBA) to inhibit ERS can significantly reverse these changes and improve muscle weight and function (78). Additionally, CHOP also disrupts the intracellular redox balance by inducing factors such as GADD34 and ERO1α, ultimately initiating a caspase-3-dependent apoptotic program that leads to the loss of muscle cells (79, 80). In diabetic animals or muscle fibers treated with PA, an increase in TUNEL-positive cells and cleaved caspase-3 protein levels can be observed, while the deletion of the CHOP gene significantly alleviates ERS-induced apoptosis (81).

It is noteworthy that the function of PERK is dual-faceted. On one hand, its signaling mediates cellular survival adaptations under stress conditions; on the other hand, persistent and dysregulated activation can drive apoptosis and atrophy (82). Studies have shown that under diabetic conditions, the activation of the PERK arm of the UPR in skeletal muscle fails or is impaired, which accelerates the collapse of protein homeostasis and the process of muscle atrophy (83). In a mouse model of diabetes with skeletal muscle-specific PERK knockout, more severe insulin resistance and muscle atrophy phenomena can be observed, which precisely demonstrates the protective role of PERK in maintaining metabolic and protein homeostasis (78). Therefore, the PERK pathway has become a key molecular bridge linking diabetic metabolic disorders, insulin resistance, and the decline in skeletal muscle mass, and fine-tuning this pathway may provide new therapeutic strategies for intervening in diabetic myopathy.

### 4.2 IRE1α

IRE1α is a highly conserved type I transmembrane sensor involved in ER stress, playing a complex and critical role in the metabolic imbalance and structural lesions of skeletal muscle in diabetes. In a diabetic environment, the metabolic disturbances trigger ERS, leading to the oligomerization and autophosphorylation of IRE1α, which activates its core endonuclease activity. The activated IRE1α produces the spliced form of XBP1 (sXBP1) through unconventional splicing, which subsequently upregulates the expression of a series of ER chaperones and genes related to ER-associated degradation, thereby enhancing the protein folding and quality control capacity of the ER, initially aimed at restoring intracellular homeostasis (84). However, when ERS persists, the IRE1α signaling shifts from adaptive to pathological. The regulatory IRE1α-dependent decay of mRNA (RIDD) it mediates not only degrades ER-related transcripts (85), but it has also been shown to target the degradation of key molecules in the insulin signaling pathway, specifically the mRNA of the insulin receptor (IR) and IRS-1, thereby exacerbating insulin resistance in skeletal muscle at the post-transcriptional level (86, 87). In skeletal muscle of diabetic model mice or myotubes treated with palmitic acid, a decrease in the mRNA levels of IR and IRS-1 can be detected. However, this degradation process is inhibited by intervention with IRE1α endonuclease inhibitors (e.g. STF-083010), leading to a partial improvement in insulin sensitivity (88).

Moreover, persistent activation of IRE1α recruits TNF receptor-associated factor 2 (TRAF2) through its intracellular domain, subsequently activating the ASK1-JNK/p38 MAPK signaling cascade (89). The sustained phosphorylation of JNK catalyzes the phosphorylation of IRS-1 at serine residues, leading to its inactivation and hindering normal insulin signal transduction (90). Simultaneously, this pro-inflammatory pathway intertwines with NF-κB signaling, collectively fostering a chronic low-grade inflammatory state in skeletal muscle, which further exacerbates insulin resistance and creates an unfavorable microenvironment for muscle maintenance (91). In transgenic mice with skeletal muscle-specific overexpression of activated IRE1α, a high activation of the JNK/NF-κB signaling pathway is observed, accompanied by severe systemic insulin resistance and reduced muscle mass (92). Dysregulation of IRE1α signaling also directly contributes to the progression of diabetic muscle atrophy. On one hand, its downstream JNK can directly phosphorylate and activate the transcription factor forkheadbox O (FoxO), which, upon entering the nucleus, upregulates the expression of E3 ubiquitin ligases Atrogin-1 and MuRF1, driving the ubiquitin-proteasome system and leading to excessive degradation of myofibrillar proteins (93). On the other hand, the IRE1α-TRAF2-ASK1 axis serves as a crucial link between ERS and cell apoptosis, promoting the activation of the mitochondrial apoptosis pathway mediated by BAX/BAK, ultimately leading to the loss of muscle cells (94). Studies have shown that in skeletal muscle of STZ-induced diabetic rats, the protein levels of IRE1α, p-ASK1, and p-JNK are synchronously elevated and significantly correlated with the upregulation of Atrogin-1 expression and the reduction of muscle fiber cross-sectional area; moreover, intervention with a function-specific inhibitor of IRE1α can effectively alleviate these pathological changes (95).

In summary, the IRE1α signaling pathway in diabetic skeletal muscle aims to adapt to stress and maintain homeostasis through the early activation of XBP1 splicing. However, prolonged activation deeply engages in the onset of insulin resistance, amplification of inflammatory responses, and execution of muscle atrophy through branches such as RIDD and JNK/p38 MAPK, making it a highly promising molecular target for intervening in diabetic myopathy.

### 4.3 ATF6

ATF6 is a crucial type II transmembrane protein in the ER stress response, whose unique non-phosphorylation activation mechanism plays an important role in the metabolic and structural remodeling of skeletal muscle in diabetes (96, 97). Under the ER stress condition induced by diabetes, ATF6 dissociates from the molecular chaperone BiP and is transported to the Golgi apparatus in a monomeric form, where it is sequentially cleaved by site 1 protease (S1P) and site 2 protease (S2P) (98). This process releases its active N-terminal fragment (ATF6N) into the cytoplasm, which acts as a transcription factor that trans locates to the nucleus to initiate the expression of a series of genes related to ER quality control, aimed at enhancing the protein folding capacity of the ER to cope with stress (99). In skeletal muscle, the activation of ATF6 is directly linked to the regulation of glucose metabolism. Studies have shown that ATF6 can induce the expression of molecular chaperones, including GRP78 and glucose-regulated protein 94 (GRP94), which play roles in maintaining the correct conformation and stability of the insulin receptor (100). In a diabetic mouse model with skeletal muscle-specific ATF6 knockout, impaired responses of key insulin signaling pathway proteins IRS-1/PI3K/Akt and reduced localization of GLUT4 on the cell membrane surface were observed, directly demonstrating the critical importance of ATF6 in maintaining insulin sensitivity in skeletal muscle (101).

In addition to metabolic regulation, the ATF6 pathway profoundly influences the quality and function of skeletal muscle. In chronic pathological conditions such as diabetes, although the initial activation of ATF6 is adaptive, its sustained activation may also be involved in the regulation of muscle atrophy. Evidence suggests that ATF6 can cooperate with other transcription factors to regulate the expression of genes associated with the autophagy-lysosomal system and the ubiquitin-proteasome system (102). Specifically, ATF6 can indirectly influence the activity of the ubiquitin-proteasome system by upregulating genes related to ER-associated degradation (ERAD) (103). More importantly, studies suggest that ATF6 can directly or indirectly regulate the expression of muscle-specific E3 ubiquitin ligases. For instance, in the context of diabetic muscle wasting, the activation of ATF6 may promote the expression of MuRF1 and Atrogin-1 by modulating the activity of the transcription factor FoxO, thereby driving protein degradation (104). In the palmitic acid-induced C2C12 myotube atrophy model, enhanced nuclear translocation of ATF6 was detected, accompanied by the accumulation of the autophagy marker LC3-II and the upregulation of E3 ligase MuRF1 expression; however, silencing ATF6 expression using siRNA significantly alleviated the activation of the aforementioned protein degradation signals and reduced myotube diameter (105). This cellular experiment directly supports the promoting role of ATF6 in muscle atrophy induced by nutrient excess. It is noteworthy that ATF6 also plays a context-dependent role in the regulation of autophagy. On one hand, as part of the adaptive UPR, ATF6 can help alleviate ERS by inducing molecular chaperones that assist in the clearance of misfolded proteins, potentially inhibiting excessive autophagy indirectly. On the other hand, under sustained and severe stress, ATF6 signaling may shift towards promoting degradation outputs. Studies have shown that ATF6 can cooperate with ATF4, which is downstream of the PERK pathway, to jointly regulate transcriptional programs that include CHOP, a known inhibitor of Bcl-2 expression that promotes Beclin-1 release, thereby activating autophagy (106). In skeletal muscle of diabetic model animals, the level of ATF6 activation is associated with changes in autophagic flux markers, suggesting its involvement in a bidirectional regulatory network of autophagy (107). Nevertheless, compared to the PERK and IRE1α pathways, the specific role of ATF6 in diabetic muscle atrophy and its downstream effect networks remain insufficiently studied, suggesting that its function may be more environmentally dependent. Research has observed that transgenic mice with skeletal muscle-specific overexpression of the active form of ATF6 exhibit improved muscle insulin sensitivity under basal conditions (108). However, under metabolic stress induced by a high-fat diet, they display more severe insulin resistance and inflammation activation. This highlights the dual nature of ATF6’s function: moderate activation helps maintain ER homeostasis and insulin signaling, while excessive or prolonged inappropriate activation may contribute to pathological processes. Therefore, future studies should utilize skeletal muscle-specific gene knockout or overexpression diabetic animal models to precisely elucidate the net effects of ATF6 under different stages and varying metabolic stresses.

**Figure 2:**
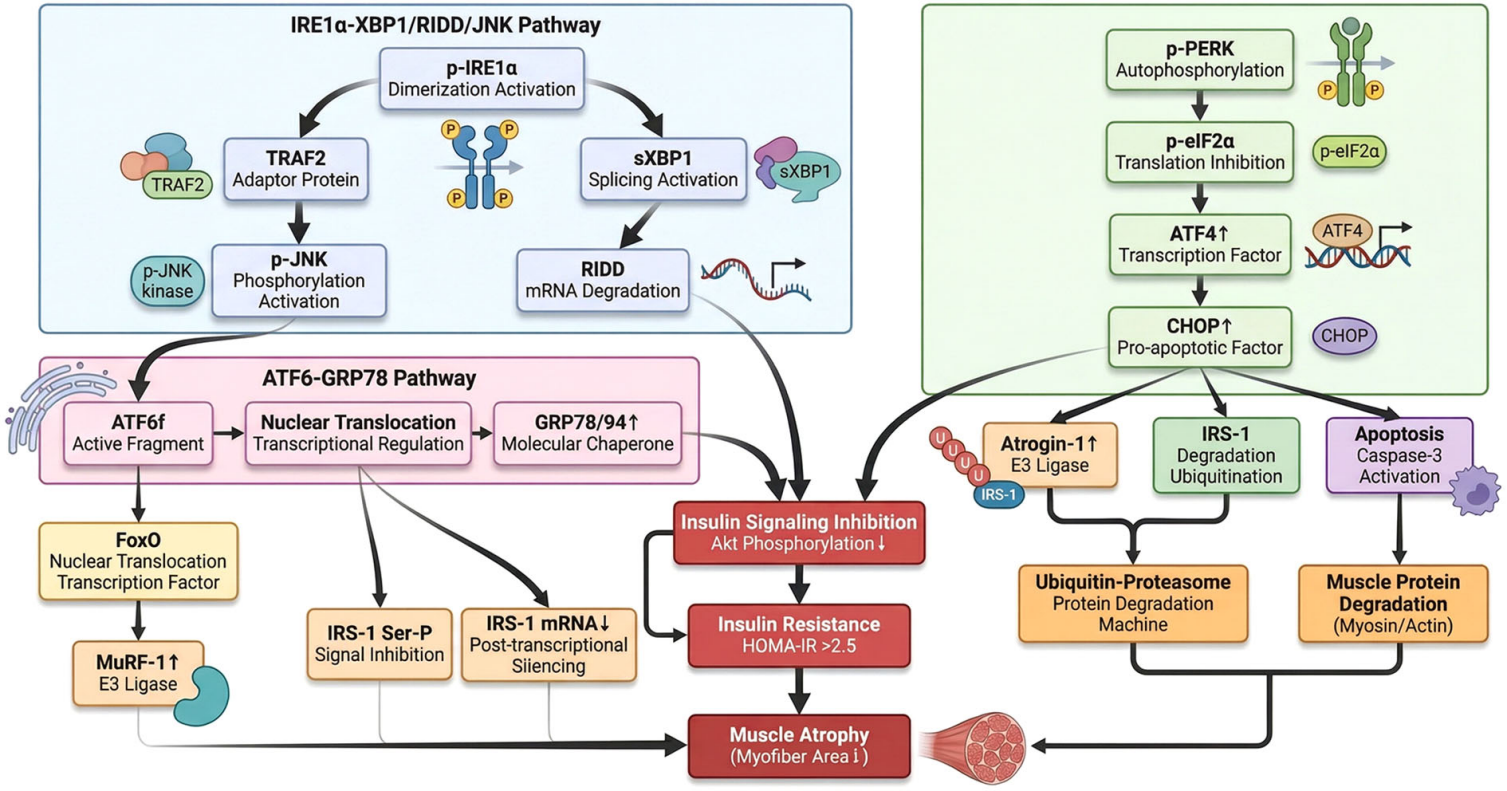
Core UPR signaling network and its downstream implications for insulin resistance and muscle atrophy. This schematic maps the three canonical UPR signaling branches and their convergent effects on skeletal muscle pathophysiology: (1) IRE1α-XBP1/RIDD/JNK Pathway: Phosphorylated (p-)IRE1α undergoes dimerization/activation, triggering two parallel cascades: (i) sXBP1 splicing (active transcription factor) and RIDD (mRNA degradation); (ii) recruitment of adaptor protein TRAF2, leading to p-JNK kinase activation. (2) PERK-eIF2α-ATF4-CHOP Pathway: Autophosphorylatedp-PERK induces p-eIF2α (translation inhibition), upregulating transcription factor ATF4 and pro-apoptotic factor CHOP. (3) ATF6-GRP78 Pathway: ERS releases active ATF6 fragment (ATF6f), which translocates to the nucleus to induce molecular chaperones (GRP78/94) and transcription factor FoxO (driving E3 ligase MuRF-1 upregulation). Downstream, UPR signaling drives insulin signaling inhibition (via reduced Akt phosphorylation, IRS-1 serine phosphorylation, and IRS-1 mRNA silencing), culminating in insulin resistance (HOMA-IR >2.5). Concurrently, UPR-mediated upregulation of E3 ligases (Atrogin-1, MuRF-1) and IRS-1 degradation promote ubiquitin-proteasome-dependent muscle protein breakdown (myosin/actin degradation) and apoptosis (caspase-3 activation), ultimately leading to muscle atrophy (reduced myofiber area).

In summary, the ATF6 pathway acts as a crucial link between ER function, insulin signaling, and muscle protein balance. Proper regulation of this pathway is vital for addressing skeletal muscle complications related to diabetes, making it a promising target for therapeutic research (**Figure 2**). Future studies should aim to clarify the specific effector molecules that operate downstream of ATF6, explore the interactions between these molecules and the PERK and IRE1α pathways, and identify effective intervention strategies at various stages of disease to maximize their protective benefits while minimizing potential negative effects.

### 4.4 Endogenous attenuation and resolution of ERS

A comprehensive understanding of ERS in T2DM myopathy should take into account not only its triggers but also the natural countermeasures that cells employ. The UPR functions not just as a warning system; it activates programs designed to actively resolve stress. Problems arise when these compensatory mechanisms become overwhelmed. Key protective strategies include the upregulation of ERAD through the IRE1α-XBP1s and ATF6 pathways, which help eliminate misfolded proteins; the induction of antioxidant enzymes, such as those activated by the PERK-ATF4 pathway or the nuclear factor erythroid 2-related factor 2 (Nrf2), to reduce ROS associated with ERS; the activation of selective autophagy (ER-phagy) to remove damaged ER; and the adaptive increase in chaperone capacity within the ER (109–111). In T2DM, the continuous metabolic stress likely exhausts these protective mechanisms, leading to a state of proteostatic collapse where the ability to repair is consistently outpaced. This perspective indicates that future therapeutic strategies may be more effective if they focus on strengthening these intrinsic resolution pathways rather than broadly inhibiting ERS.

## 5 Interrelationships between ERS and other T2DM-related pathogenic mechanisms

### 5.1 ERS and mitochondrial dysfunction

ERS and mitochondrial dysfunction constitute the core pathological basis of skeletal muscle insulin resistance in T2DM through precise molecular dialogue. This process begins with metabolic stress due to nutrient excess and is amplified through intricate molecular interactions at the MERC.

The primary sensor of ERS, PERK, upon activation, mediates the phosphorylation of eIF2α to regulate protein translation and directly phosphorylates specific serine residues on IRS-1 (e.g. Ser312) (112). This specific phosphorylation event directly hinders the binding of IRS-1 to the IR and disrupts the interaction between IRS-1 and the p85 regulatory subunit of phosphoinositide 3-kinase (PI3K), thereby fundamentally interrupting the transmission of insulin signaling (113). Meanwhile, another branch of the ERS pathway, IRE1α, recruits TRAF2, which in turn activates c-Jun N-terminal kinase (JNK). The activated JNK also phosphorylates the serine sites of IRS-1, further exacerbating the inhibition of insulin signaling and forming a synergistic destructive effect with the PERK pathway (114, 115).

At the molecular level, the Mitochondria-Associated Endoplasmic Reticulum Membranes (MAM) serve as a hub for information exchange between the ER and mitochondria (116, 117). The IP3R located on the ER membranes forms a stable complex with the voltage-dependent anion channel 1 (VDAC1) situated on the outer mitochondrial membrane through the molecular chaperone GRP75 (118, 119). Under ERS conditions, the calcium stores of the ER are excessively released, causing calcium ions to uncontrollably surge into the mitochondria via the IP3R-GRP75-VDAC1 channel. This calcium overload directly inhibits the activity of pyruvate dehydrogenase (PDH), impairing the tricarboxylic acid cycle; simultaneously, it excessively activates dehydrogenases in the tricarboxylic acid cycle and complex I of the electron transport chain, leading to increased electron leakage and a substantial generation of superoxide and other ROS (120, 121). Furthermore, calcium ions also act as a cofactor for nitric oxide synthase (NOS), and elevated concentrations promote the production of reactive nitrogen species (RNS), which, together with ROS, exacerbate oxidative stress (122).

Mitochondrial ROS specifically target the insulin signaling pathway: Mitochondrial-derived ROS are not non-specific damage. They can specifically oxidize and inhibit the phosphorylation and kinase activity of AKT (PKB) (123). AKT is a critical node downstream of PI3K in the insulin signaling pathway, and its inactivation directly leads to a sustained inhibitory state of the AS160/TBC1D4 protein, thereby preventing the translocation of GLUT4 vesicles to the cell membrane and ultimately closing the door to glucose uptake (124). Furthermore, ROS can activate the mTORC1/S6K1 pathway, which, once activated, can feedback phosphorylate other serine sites on IRS-1, forming another negative feedback loop that disrupts insulin signaling (125, 126).

The interaction between energy sensing and mitochondrial UPR occurs when a decline in ATP production, resulting from mitochondrial dysfunction, activates the energy sensor AMPK (127). While acute activation of AMPK is beneficial, in a chronic energy crisis state, it competes with the insulin signaling pathway for cellular resources. More importantly, the abnormal proteins or peptides produced by damaged mitochondria can induce the mitochondrial UPR, with key mediators such as CHOP and ATF4 being upregulated. These transcription factors can directly regulate the expression of genes associated with insulin resistance and inflammation, such as enhancing the expression of suppressor of cytokine signaling 3 (SOCS3), which accelerates the proteasomal degradation of IRS-1 by competitively binding to IR (128, 129).

In summary, in skeletal muscle of T2DM, ERS and mitochondrial dysfunction directly attack insulin signaling molecules via the PERK/IRE1α-JNK pathway, disrupt metabolic homeostasis and redox balance through the calcium- ROS axis mediated by MERC, and engage in transcriptional reprogramming through the mitochondrial UPR and energy sensing pathways. These precise molecular events intertwine to form a dense network that ultimately culminates in the failure of the IRS-1/PI3K/AKT signaling axis, collectively orchestrating the pathological process of insulin resistance in skeletal muscle. Interventions targeting these specific molecular nodes, such as PERK-specific inhibitors or MERC stabilizers, are emerging as highly promising therapeutic strategies.

### 5.2 ERS and autophagy

In the intricate metabolic milieu of T2DM, skeletal muscle tissue encounters metabolic stress induced by sustained hyperglycemia, elevated levels of free fatty acids, and insulin resistance (130). This chronic state of stress disrupts ER homeostasis, leading to the activation of ERS. Notably, ERS demonstrates a dual role in the context of muscle wasting in T2DM; it initially functions as an adaptive protective mechanism but ultimately evolves into a significant mediator of tissue degradation. Recent investigations have revealed that the interplay between ERS and autophagy constitutes a complex regulatory network, and an imbalance within this network is a fundamental contributor to the disruption of skeletal muscle protein homeostasis, culminating in the loss of myofibers (131).

#### 5.2.1 Molecular pathways of ERS-regulated autophagy

The PERK pathway is the most clearly defined signaling route that connects the ER stress response with autophagy. Activated PERK mediates the selective translation of the transcription factor ATF4 by phosphorylating eIF2α (132). ATF4 not only upregulates the transcription of core autophagy genes such as autophagy-related gene 5 and microtubule-associated protein 1 light chain 3 (LC3), but also regulates the autophagic process in conjunction with CHOP by forming a transcriptional complex (133). Research has shown that in skeletal muscle of diabetic models, the co-localization of ATF4 and CHOP is significantly positively correlated with the expression of the autophagy marker LC3-II (134). The activation of the IRE1α pathway recruits TRAF2 through its intracellular kinase domain, thereby activating JNK (135, 136). Activated JNK promotes the dissociation of Bcl-2 from Beclin-1 by phosphorylating multiple serine residues on Bcl-2, releasing Beclin-1 to initiate autophagosome formation (137, 138). Furthermore, the endonuclease activity of IRE1α can selectively splice mRNA in an atypical manner, further expanding its regulatory scope over the autophagy network (139). Compared to the previous two pathways, ATF6’s regulation of autophagy is more indirect. ATF6f, as a transcription factor, primarily regulates the expression of the ER molecular chaperone GRP78 and genes related to ERAD. When the adaptive response is insufficient to alleviate stress, ATF6f may synergize with the PERK and IRE1α pathways by regulating genes related to lysosomal biogenesis (140, 141).

#### 5.2.2 Pathophysiological significance of the ERS-autophagy axis

In the context of T2DM, persistent metabolic stress leads to a transition of ERS signaling from adaptive to apoptotic. Among these, the sustained high expression of the transcription factor CHOP is considered a key molecular event in this transition. In addition to promoting the release of Beclin-1 through the classical pathway of downregulating Bcl-2 expression, CHOP can also directly regulate the epigenetic status of autophagy-related genes. Recent studies have gradually revealed that the interaction between organelles plays a crucial role in ERS-mediated muscle atrophy (142, 143). MAMs serve as platforms for signaling, where the Syntaxin4 (STX4) protein, located on the outer membrane of mitochondria, has been confirmed as an important factor in maintaining skeletal muscle mitochondrial homeostasis (144, 145). Research has shown that in skeletal muscle-specific STX4 knockout models, significant mitochondrial fragmentation, abnormal electron transport chain function, and impairment of the PINK1/PARKIN-mediated mitochondrial autophagy pathway are observed (144). Another noteworthy direction is the role of peroxisomes in the ERS-autophagy axis. Recent studies indicate that the skeletal muscle-specific knockout of the peroxisome biogenesis factor peroxisomal biogenesis factor 5 leads to severe mitochondrial dysfunction and muscle atrophy (146, 147). Notably, a similar decline in peroxisomal function has also been observed in naturally aged muscle tissues, suggesting that this may be a common mechanism underlying metabolic-related muscle atrophy (148).

#### 5.2.3 The vicious cycle: impaired autophagy exacerbates ERS

The reciprocal exacerbation between impaired autophagy and ERS forms a self-amplifying vicious cycle, driving proteostasis collapse in diabetic skeletal muscle. While ERS can disrupt autophagic flux, conversely, deficient autophagic clearance—due to nutrient-sensing dysregulation (149, 150), aging (151), or as a consequence of ERS itself—leads to the accumulation of dysfunctional organelles and protein aggregates. A pivotal mechanism is defective mitophagy, which results in elevated mitochondrial ROS production (152). This ROS can directly inhibit SERCA activity (153), disrupting ER calcium homeostasis and thereby initiating or aggravating ERS. Concurrently, inadequate clearance of stressed ER fragments via ER-phagy may perpetuate the activation of UPR sensors (154, 155). Thus, within the diabetic milieu, ERS and autophagy dysfunction engage in a feed-forward loop: metabolic stress induces ERS, which impairs autophagy; the resulting autophagic failure further increases proteotoxic burden and organellar stress, leading to intensified and sustained ERS. This pathophysiological framework implies that interventions restoring autophagic function could concurrently mitigate ERS (156).

### 5.3 ERS and inflammation

ERS not only directly induces the production of inflammatory mediators but also creates a self-reinforcing feedback loop that continuously disrupts muscle homeostasis. The interplay between this inflammatory response and ER stress exacerbates the pathological progression of T2DM, leading to increased muscle atrophy and functional impairment.

#### 5.3.1 Molecular mechanisms of ERS-induced inflammatory response

The core mechanism of ER stress-induced inflammatory response involves the coordinated activation of three signaling pathways of the unfolded protein response. In the IRE1α branch, activated IRE1α recruits TRAF2 through its cytoplasmic domain, forming the IRE1α-TRAF2 complex. This complex acts as a molecular scaffold that further recruits and activates the IKK complex, leading to the phosphorylation and degradation of IκBα protein, which releases NF-κB and drives its nuclear translocation (156). Notably, recent studies have found that the ribonuclease activity of IRE1α can regulate the stability of specific inflammation-related mRNAs through an atypical splicing mechanism, a process referred to as RIDD, which may play an important role in the fine-tuning of the inflammatory response (157).

The PERK-ATF4-CHOP signaling axis is also crucial in the ERS-mediated inflammatory response. Phosphorylated PERK directly binds to the promoter regions of various inflammatory mediator genes through its downstream effectors ATF4 and CHOP (158). Notably, CHOP has been found to directly regulate the expression of the NLRP3 inflammasome and indirectly enhance the amplification of inflammatory signals by promoting the production of ROS (159). The absence of CHOP significantly attenuates the activation of the NLRP3 inflammasome and reduces the cleavage levels of caspase-1, suggesting that CHOP plays a critical bridging role between ERS and inflammasome activation (160). Although the ATF6 pathway is relatively indirect, its mediated transcriptional reprogramming also participates in inflammatory regulation. Activated ATF6 indirectly affects the activation state of NF-κB by upregulating the expression of the ER molecular chaperone GRP78 (141). Recent studies have shown that ATF6 can also cooperate with XBP1s to regulate the expression of components of the ERAD pathway, thereby affecting the duration and intensity of inflammatory signals. From the perspective of organelle interactions, recent research has revealed the critical role of MAMs in ERS-induced inflammation (161). The mitochondrial antiviral signaling protein (MAVS) located at MAMs has been found to serve as an integration platform for IRE1α and PERK signaling, facilitating the cascade amplification of inflammatory signals (162). In diabetic skeletal muscle, the phosphorylation level of MAVS is significantly elevated, and its interaction with IRE1α is enhanced, suggesting the important role of MAMs remodeling in the ERS-related inflammatory response (78). The epigenetic regulatory mechanisms have also received widespread attention in recent years. Studies have found that sustained ERS can influence the expression of inflammatory genes by altering histone modification patterns. For instance, ATF4 has been shown to recruit the histone acetyltransferase p300 to the promoter regions of the IL-6 and IL-8 genes, promoting histone H3K27 acetylation, thereby establishing an open chromatin conformation that enhances the transcriptional activity of inflammatory genes (163).

#### 5.3.2 Reverse regulation of inflammatory signals on ERS

Inflammatory signals constitute a sophisticated feedback amplification network that inversely regulates ERS. TNF-αcan exacerbate ERS through multiple mechanisms after activation via its type I receptor (TNFR1) (164). Studies have shown that TNF-α promotes the generation of ceramide by activating neutral sphingomyelinase, which in turn disrupts ER calcium homeostasis and increases the protein folding burden (164). Additionally, TNF-α can downregulate the expression of SERCA2b through the activation of the NF-κB signaling pathway, impairing the endoplasmic reticulum’s calcium ion uptake capacity and further compromising the protein folding environment (165). IL-1β regulates ERS primarily through the JNK-p38 MAPK signaling axis (166). Activated JNK phosphorylates the ER transmembrane protein BAP31, affecting its interaction with procaspase-8, thereby altering the sensitivity of the ERS sensor (167).

Recent studies have found that inflammatory signals can also influence ERS levels by regulating the ERAD system (168). TNF-α has been shown to inhibit the expression of the key ERAD component HRD1, leading to the accumulation of misfolded proteins in the ER lumen, which further exacerbates ERS (169, 170). The mature IL-1β and IL-18 produced after the activation of the NLRP3 inflammasome not only affect ERS through classical receptor signaling but also directly interfere with ER function during their assembly process (171). Research has revealed that the NLRP3 inflammasome component ASC can form a complex with ERS sensors, altering the output characteristics of the UPR signaling (172). Inflammatory signals indirectly affect ERS by altering cellular energy metabolism states. Pro-inflammatory factors can induce an increase in glycolytic flux in skeletal muscle cells, leading to lactic acid accumulation and a decrease in intracellular pH, which in turn affects the protein folding efficiency within the ER lumen. Additionally, mitochondrial dysfunction caused by inflammatory signals results in insufficient ATP supply, further impairing the ATP-dependent protein folding processes in the ER (173). Recent single-cell transcriptomic studies have revealed the cellular heterogeneity of the inflammatory-ERS feedback loop (174). In diabetic skeletal muscle, different types of muscle fibers exhibit significant differences in their responses to inflammatory signals, with type II fast-twitch muscle fibers showing higher sensitivity to ERS, which may be one of the important reasons for their greater propensity to undergo atrophy (78).

In addition, the role of non-coding RNAs in this regulatory network is gradually being revealed. miR-146a has been found to play a key role in the inflammation-ERS feedback loop, with its expression regulated by NF-κB, while it can also target the 3’UTR regions of IRE1α and PERK, forming a finely tuned feedback regulation (175, 176). Circular RNA circPHKA2 has been shown to regulate the expression of GRP78 by adsorbing miR-1254, participating in the regulation of ERS by inflammatory signals (177).

Collectively, these studies delineate the interaction mechanisms between inflammation and ERS and pinpoint multiple potential targets for intervening in T2DM-associated muscle atrophy. Future research needs to further elucidate the spatiotemporal dynamics of these complex regulatory networks and their specific manifestations in different muscle cell types.

## 6 Therapeutic prospects: targeting ERS for T2DM myopathy

### 6.1 Therapy targeting the PERK pathway

The PERK-eIF2α-ATF4-CHOP pathway is one of the core branches of the ERS response. Its excessive activation can inhibit protein synthesis and induce apoptosis, playing a critical role in T2DM myopathy. Various components found in chemical chaperones and natural compounds can specifically target this pathway to exert therapeutic effects.

4-Phenylbutyric acid (4-PBA), as a broad-spectrum chemical chaperone, significantly reduces fasting blood glucose and reverses the decline in skeletal muscle weight in db/db T2DM model mice when administered orally at a dose of 1 g/kg/day. Mechanistically, 4-PBA reduces protein synthesis inhibition and apoptosis by directly inhibiting the activation of the PERK-eIF2α-ATF4-CHOP pathway (178). Moreover, proteomic analysis reveals that 4-PBA can restore damaged mitochondrial function in diabetic skeletal muscle, including enhancing the activity of mitochondrial respiratory chain complexes I and IV and increasing PGC-1α expression, which partially corrects the ERS-mitochondrial coupling dysfunction (179). Notably, as a weak histone deacetylase (HDAC) inhibitor, 4-PBA upregulates the expression of endoplasmic reticulum-resident molecular chaperones and antioxidant genes by altering histone acetylation status, thereby expanding its functions beyond traditional chemical chaperones at an epigenetic level (180, 181).

Ginsenoside Compound K (CK), as a natural compound, significantly alleviates the decrease in limb muscle weight in a high-fat diet combined with streptozotocin-induced T2DM mouse model when administered daily at a dose of 20 mg/kg for 8 weeks. The study indicates that CK effectively reduces the expression of phosphorylated PERK, phosphorylated eIF2α, and the downstream pro-apoptotic protein CHOP in the gastrocnemius muscle, directly inhibiting the PERK pathway (182). Additionally, CK activates the Nrf2 signaling pathway by disrupting the Keap1-Nrf2 interaction, promoting the expression of antioxidant genes such as heme oxygenase-1 (HO-1), thereby further alleviating ERS (183). However, the therapeutic targeting of the PERK pathway must contend with a fundamental pharmacological challenge: the dual nature of the UPR. Broad-spectrum agents like 4-PBA, while effectively suppressing chronic, maladaptive signaling (e.g., the PERK-ATF4-CHOP axis), may simultaneously blunt the initial, adaptive arm of the UPR. This adaptive response is physiologically crucial for restoring proteostasis and promoting cell survival under transient stress. Indiscriminate inhibition could therefore, in theory, impair the muscle’s innate capacity to manage metabolic fluctuations, potentially yielding unintended negative consequences for muscle homeostasis. This risk highlights the need to develop branch-specific or context-dependent modulators. The ideal pharmacological approach would selectively reduce sustained pro-apoptotic signals while preserving or enhancing early pro-survival adaptive responses. Future research should focus on identifying agents with greater specificity for maladaptive UPR branches and exploring timing-based therapeutic windows.

In summary, the therapeutic potential of targeting the PERK pathway with agents like 4-PBA and ginsenoside CK remains primarily supported by preclinical evidence. Although 4-PBA has an established safety profile in humans, owing to its FDA approval for urea cycle disorders, this does not guarantee efficacy in the context of diabetic myopathy, which awaits clinical validation (184). Similarly, the promising results of ginsenoside CK from animal studies are constrained by unanswered questions regarding its pharmacokinetics and long-term safety in patients. Beyond efficacy, critical translational hurdles for both compounds include poor tissue specificity and suboptimal bioavailability. Future research should therefore focus on overcoming these barriers through strategies such as developing muscle-targeted delivery systems (e.g., nanoparticle-based carriers) or evaluating synergistic effects in combination with established glucose-lowering therapies (e.g., metformin).

### 6.2 Therapy targeting the IRE1α pathway

The IRE1α pathway is another core branch of the ER stress response. Its excessive activation can inhibit insulin signaling and promote apoptosis through the IRE1α-JNK signaling pathway, leading to muscle atrophy in T2DM myopathy. Various compounds can directly target this pathway to restore insulin sensitivity and muscle homeostasis.

Tauroursodeoxycholic acid (TUDCA), as a chemical chaperone, significantly improves systemic glucose homeostasis and insulin sensitivity in a mouse model of obesity and insulin resistance induced by a high-fat diet following continuous intraperitoneal injection of TUDCA (250 mg/kg/day for 4 weeks) (185). In skeletal muscle, TUDCA effectively inhibits the excessive activation of the IRE1α branch, as evidenced by a significant reduction in phosphorylated IRE1α (p-IRE1α) and its downstream target p-JNK levels (186). The inhibition of JNK activity reduces its serine phosphorylation of IRS-1, thereby restoring the functionality of the insulin signaling pathway (including Akt phosphorylation). Additionally, TUDCA reduces the expression of the pro-apoptotic factor CHOP in the gastrocnemius and soleus muscles. This reduction is associated with an increase in the cross-sectional area of muscle fibers and the preservation of muscle wet weight. These findings support the notion that TUDCA prevents diabetic muscle atrophy through two mechanisms: enhancing insulin signaling and inhibiting apoptosis mediated by ERS (187).

Tangeretin, as a natural polymethoxyflavone, directly targets the IRE1α signaling branch in the palmitic acid-induced C2C12 myotube ER stress model at a concentration of 20 μM (188). Molecular docking simulations and cellular thermal shift analyses indicate that tangeretin binds to specific domains of IRE1α, inhibiting its stress-induced oligomerization and autophosphorylation, thereby weakening its RNase activity, which results in a significant reduction in the mRNA and protein levels of the spliced form of XBP1 (XBP1s) (189). As the core effector molecule of IRE1α/XBP1 pathway, sXBP1 plays an important role in diabetic myopathy. sXBP1 can enhance the protein folding ability of endoplasmic reticulum and alleviate ERS by up-regulating the expression of molecular chaperones GRP78 and ERAD-related genes (190). Clinical studies have found that although sXBP1 expression is increased in skeletal muscle of diabetic patients, the transcriptional activity of its downstream protective genes is limited, which may be related to the interference of oxidative stress and inflammatory factors (78). However, the bioavailability issues of TUDCA and tangeretin still need to be addressed, and future efforts should focus on optimizing delivery systems and clinical validation.

Targeting the IRE1α pathway offers a dual therapeutic prospect for diabetic myopathy: restoring insulin signaling and attenuating pro-apoptotic pathways in skeletal muscle. Translational support emerges from studies on TUDCA. Beyond its established use in hepatobiliary disorders, a randomized controlled trial in obese, insulin-resistant subjects demonstrated that TUDCA treatment (1,750 mg/day for 4 weeks) significantly improved hepatic and muscle insulin sensitivity and enhanced insulin-stimulated IRS-1/Akt phosphorylation in muscle, providing key proof-of-concept in humans (191). However, this study did not assess muscle mass or strength, leaving its direct efficacy against muscle atrophy unconfirmed. In contrast, natural compounds like tangeretin remain in the preclinical domain, with unresolved pharmacokinetic challenges. A fundamental hurdle for IRE1α-directed therapy is achieving sufficient selectivity to inhibit maladaptive RIDD/JNK outputs while preserving adaptive XBP1s signaling. Therefore, future efforts must not only evaluate repurposed agents like TUDCA in trials with robust musculoskeletal endpoints but also advance the development of next-generation, pathway-selective IRE1α modulators.

### 6.3 Therapy targeting the ATF6 pathway

The ATF6 pathway regulates the ER’s folding capacity during ERS. Its adaptive activation can enhance protein processing and prevent apoptosis, showing potential in the prevention and treatment of T2DM myopathy. Lifestyle interventions, such as exercise, can specifically target this pathway, inducing beneficial stress responses.

Exercise interventions, particularly resistance training, have been shown to preferentially activate the ATF6 pathway, which results in an adaptive UPR. In human studies, a single session of resistance exercise in healthy subjects resulted in increased nuclear ATF6 levels observed in vastus lateralis muscle biopsies, accompanied by upregulation of its target genes, such as protein disulfide isomerase (PDI) and ER DNA J domain-binding protein 1, thereby enhancing ER folding capacity without activating the PERK-eIF2α-CHOP pathway or apoptotic signals (192). This hormonal effect prepares muscles for subsequent metabolic stress. Furthermore, exercise induces the secretion of fibroblast growth factor 21, which improves muscle insulin sensitivity and ERS through autocrine/paracrine mechanisms. Additionally, FGF21 promotes the “browning” of white adipose tissue via endocrine actions, thereby contributing to a reduction in metabolic stress in skeletal muscle (193).

Time-restricted feeding, as a chronobiological intervention, indirectly regulates ATF6-related UPR genes by aligning the feeding window with biological clock rhythms. In skeletal muscle, core clock proteins (such as BMAL1/CLOCK) directly regulate the rhythmic expression of UPR genes, including Bip and Chop (194). Studies investigating the effects of high-intensity interval training (HIIT) combined with TRF on T2DM rats demonstrate that the combined treatment (D-T+HIIT group) is more effective in improving blood glucose, insulin resistance, and markers of muscle degradation, while also activating AKT, mTOR signaling, and IGF-1 expression, yielding results comparable to the non-diabetic group. The combined intervention also enhances muscle fiber cross-sectional area and muscle tissue morphology, indicating a synergistic effect of HIIT and TRF in promoting muscle protein synthesis and glucose regulation (195).

**Figure 3:**
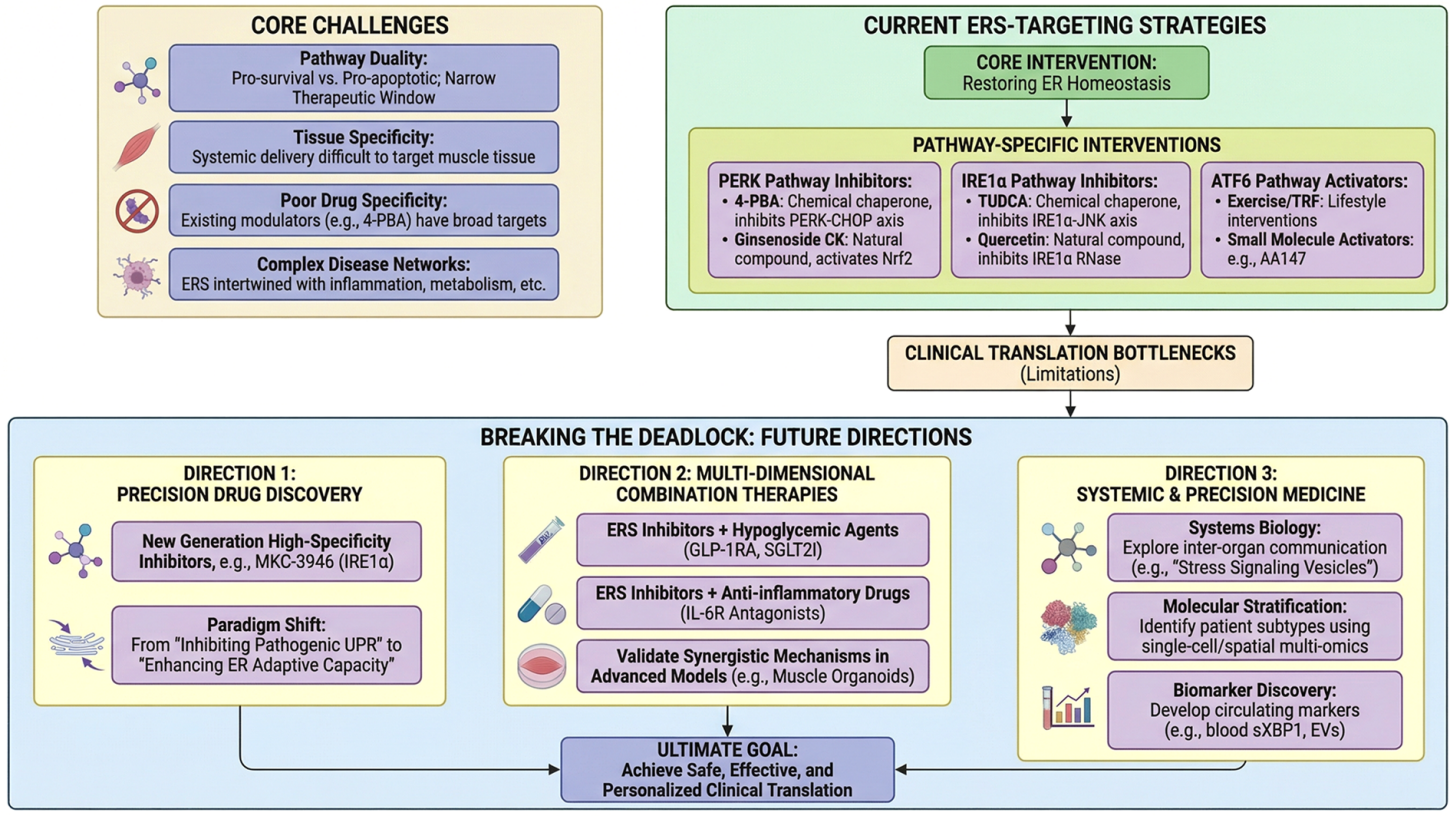
ERS-targeted therapy: Challenges, strategies, and future directions. This diagram summarizes barriers and solutions for ERS-targeted interventions in muscle metabolic disorders: Core challenges: Narrow therapeutic window, poor tissue/drug specificity, and ERS-inflammation/metabolism crosstalk. Current strategies: Pathway-specific agents (e.g., 4-PBA for PERK, TUDCA for IRE1α) to restore ER homeostasis. Future directions: (1) Precision drugs (e.g., MKC-3946); (2) Combination therapies (ERS inhibitors + hypoglycemic/anti-inflammatory agents); (3) Precision medicine (systems biology, single-cell omics, sXBP1 biomarkers). Goal: Safe, personalized clinical translation of ERS-targeted treatments.

The individualization and precision of lifestyle interventions represent the future direction in managing T2DM. It is essential to clarify the optimal regulatory patterns of different exercise modalities (e.g. resistance versus aerobic) and nutritional strategies (e.g. time-restricted feeding) on skeletal muscle energy regulatory systems in specific T2DM populations. Furthermore, employing multi-omics technologies to identify predictive biomarkers will be crucial for optimizing non-pharmacological prescriptions (**Figure 3**).

## 7 Limitations and future directions

While the targeting of ERS presents a compelling therapeutic rationale for T2DM myopathy, its journey from bench to bedside remains fraught with challenges. The promising preclinical results for compounds like 4-PBA and TUDCA, detailed in the previous section, have yet to be substantiated in human trials. Translating this mechanistic knowledge into clinical practice urgently requires parallel progress on two fronts: identifying robust biomarkers of muscle-specific ERS activity in patients, and designing clinical trials that are informed by this pathophysiology. Here, we outline the key translational gaps and propose a roadmap for future research.

### 7.1 The critical need for clinical biomarkers

The clinical translation of ERS-targeted therapies for T2DM myopathy is hampered by a lack of biomarkers that can specifically and reliably measure endoplasmic reticulum stress within skeletal muscle. To effectively identify patients who might benefit from such interventions and to evaluate treatment efficacy in trials, the development of specific biomarkers is essential.

Currently available candidates, however, are inadequate. The circulating level of spliced XBP1 (sXBP1), though directly linked to IRE1α activation, reflects total systemic ER stress rather than changes specific to muscle tissue. Other potential markers like GRP78 and phosphorylated PERK present even greater challenges for clinical use. Their fundamental limitation is a lack of tissue specificity, as elevated levels in blood could originate from multiple organ systems under metabolic stress, not solely from skeletal muscle. This is compounded by technical constraints; common assays often lack the sensitivity to detect the subtle increases expected from a single tissue against the high background in biofluids. Pre-analytical factors further confound measurements, as biomarker levels are highly sensitive to sample handling, processing delays, and sample type. Beyond these technical issues, the biological context of T2DM itself complicates interpretation. Chronic disease adaptation and common comorbidities such as kidney disease or systemic inflammation can alter the circulating levels of these proteins independently of muscle ERS. Most importantly, there are no clinically validated thresholds or standardized methods to interpret these measurements for T2DM myopathy, rendering them unsuitable for patient stratification or monitoring.

Future progress requires a concerted effort to link deep molecular analysis of muscle tissue from biopsies with comprehensive profiling of blood samples. This integrated approach may yield novel, muscle-derived signals in the circulation with the necessary specificity. Until such markers are established, clinical trials of ERS modulators should consider including direct molecular assessment of muscle biopsies as a primary pharmacodynamic readout, supplemented by exploratory analysis of circulating markers.

### 7.2 Pathways to clinical validation

No ERS-targeted drug is currently approved for diabetic myopathy. A pragmatic translational strategy may involve drug repurposing or rational combination therapy. For example, TUDCA has a known safety profile from trials in other diseases (196). Combining such an agent with a standard glucose-lowering drug (e.g. an SGLT2 inhibitor) could simultaneously address systemic metabolism and cellular stress (197–199). Future clinical trials must be carefully constructed: (1) Patient selection should enrich for participants likely to respond, potentially using biomarker signatures suggestive of active muscle ERS; (2) Meaningful endpoints must include not only glycemic control but also improvements in muscle health-assessing mass (via MRI/DXA), strength (e.g. grip strength), and physical function (e.g. gait speed); and (3) Given the UPR’s dual role, vigilant safety monitoring is essential in long-term studies to guard against unintended disruptions to normal proteostatic function.

### 7.3 Embracing systemic complexity and combination approaches

The multifactorial nature of T2DM myopathy calls for combination strategies. Pairing an ERS modulator with an anti-inflammatory agent (e.g. an IL-6 pathway inhibitor) could block complementary pathological loops. Furthermore, we must consider skeletal muscle as part of a dynamic system. Emerging data suggest that stressed metabolic organs secrete extracellular vesicles carrying UPR-related signals (e.g. phosphorylated IRE1α), which can remotely influence muscle (200). Understanding this inter-organ crosstalk opens new avenues for systemic intervention.

### 7.4 Concluding perspective: from mechanism to patient

In summary, recognizing the therapeutic potential of ERS modulation requires a comprehensive translational pipeline. Key focus areas include the discovery and validation of precision biomarkers through advanced profiling of human tissues. Additionally, it is important to conduct clinically guided trials that are informed by underlying mechanisms and relevant functional outcomes. Finally, exploring the systemic biology of organ communication is crucial. By integrating these elements, research can transition from generalized approaches to personalized interventions, ultimately aiming to reduce muscle decline in individuals with T2DM.

## 8 Conclusion

This review focuses on the crucial cellular stress response known as ERS, which is triggered by factors such as hyperglycemia, lipotoxicity, and inflammation. ERS serves as a central component in the pathology of T2DM. By synthesizing recent experimental studies, we clarify how ERS disrupts muscle homeostasis through UPR pathways, specifically involving PERK, IRE1α, and ATF6. These disruptions contribute significantly to the development of insulin resistance and the activation of protein degradation systems. Given the evidence implicating ERS in T2DM-related muscle disorders, therapeutic strategies aimed at modulating ERS represent a promising avenue for investigation. However, translating this approach into effective clinical interventions requires a balanced targeting of its adaptive and terminal pathways, which may vary across patient populations. However, effectively regulating its dual roles is challenging. Adaptive responses promote cell survival, while terminal responses can lead to apoptosis and functional decline. Therefore, it is essential for researchers to find a balance between these opposing responses to develop effective interventions for patients with T2DM.We advocate for more comprehensive investigations into the regulatory mechanisms governing ERS signaling pathways. Such research could lead to the formulation of more precise therapeutic strategies. Additionally, establishing robust animal models and conducting detailed clinical trials are critical for validating the efficacy of various interventions across diverse patient populations. Ultimately, this review emphasizes the importance of understanding how ERS contributes to T2DM myopathy. By identifying potential therapeutic targets, we lay the groundwork for future clinical research aimed at developing effective interventions. As we explore the complexities of ERS in relation to T2DM, focusing on innovative strategies will be crucial for improving muscle health and enhancing the overall quality of life for affected patients.

## Glossary

4-PBA: 4-phenylbutyric acid
AGEs: Advanced glycation end products
Akt: Protein kinase B
AMPK: AMP-activated protein kinase
ASK1: Apoptosis signal-regulating kinase 1
ATF4: Activating transcription factor 4
ATF6: Activating transcription factor 6
BIP: Binding immunoglobulin protein
CAT: Catalase
CHOP: C/EBP homologous protein
eIF2α: Eukaryotic initiation factor 2 alpha
ER: Endoplasmic reticulum
ERO1α: Endoplasmic reticulum oxidoreductin1 alpha
ERAD: ER-associated degradation
FoxO: Forkheadbox O
GLUT4: Glucose transporter type 4
GRP78: Glucose-regulated protein 78
GRP94: Glucose-regulated protein 94
HbA1c: Hemoglobin A1c
HIIT: High-intensity interval training
HOMA-IR: Homeostasis model assessment of insulin resistance
IL: Interleukin
IP3R: Inositol trisphosphate receptor
IRE1α: Inositol-requiring enzyme 1 alpha
IRS-1: Insulin receptor substrate 1
JNK: c-Jun N-terminal kinase
LPO: Lipid peroxides
MAVS: Mitochondrial antiviral signaling protein
MERC: Mitochondria-ER contact sites
MAMs: Mitochondria-Associated (Endoplasmic Reticulum) Membranes
mTORC1: Mechanistic target of rapamycin complex 1
MuRF1: Muscle RING finger protein 1
NOS: Nitric oxide synthase
Nrf2: Nuclear factor erythroid 2-related factor 2
RIDD: Regulated IRE1-Dependent Decay
ROS: Reactive oxygen species
S1P: Ssite-1 protease
S2P: Site-2 protease
SERCA: Sarco(endo)plasmic Reticulum Ca²⁺-ATPase
SOD: Superoxide dismutase
sXBP1: Spliced X-box binding protein 1
TNF: Tumor necrosis factor
TRAF2: TNF receptor-associated factor 2
TRB3: Tribbles homolog 3
TRF: Time-restricted feeding
TUDCA: Tauroursodeoxycholic acid
UPR: Unfolded Protein Response
VDAC1: Voltage-dependent anion channel 1
